# Editorial: The Present and Future of Immunology Education

**DOI:** 10.3389/fimmu.2021.744090

**Published:** 2021-08-02

**Authors:** Andrea Bottaro, Deborah M. Brown, John G. Frelinger

**Affiliations:** ^1^Department of Biomedical Sciences, Cooper Medical School of Rowan University, Camden, NJ, United States; ^2^Department of Viral Immunology, Trudeau Institute, Saranac Lake, NY, United States; ^3^Department of Microbiology and Immunology and the Wilmot Cancer Center, University of Rochester School of Medicine and Dentistry, Rochester, NY, United States

**Keywords:** immunology, medical, active, learning, education, undergraduate, diversity, laboratory

## Introduction

This Research Topic addresses issues relevant to teaching of modern immunology, a field that has exploded in recent years and is constantly evolving. These articles encompass curricular innovation, new pedagogical strategies, and teaching tools for the current and future generations of immunologists. The wide range of articles in this Research Topic illustrate diverse approaches for teaching basic tenets of immunology. Fortunately, immunologists are well-acquainted with diversity. Channeling one of the basic principles of Immunology, it is hoped the readers of this issue will select the Immunology articles that are most helpful to their particular mode of teaching. The articles presented are aimed at practitioners -the faculty members who are teaching and organizing Immunology courses. One of the goals of this issue is to give concrete examples of teaching strategies and concepts that could be modified for their own particular situation. In immunologic terms, this could be thought of as affinity maturation: starting with the ideas presented here, approaches can be actively fine-tuned for the particular situation of the faculty preceptor. Another major aspect of diversity illustrated in the articles presented is that students vary immensely in terms of their background knowledge. There are also fundamentally different educational systems in different countries. Finally, the articles reflect inherent constraints of the course such as the time allotted for the class that impact what is possible to cover in Immunology courses. Indeed, perhaps the major challenge in designing courses is not finding important Immunology subjects amid the myriad of interesting topics to cover (1) but rather what to leave out. We have grouped this editorial into sections for the convenience of the reader with particular interests, but it is important to note that the approaches are more general and can be used at different levels of teaching. While these articles highlight the many different ways to approach teaching immunology, they all reflect the enthusiasm and diversity of the faculty who teach immunology.

## Diversity of Approaches in Undergrad Teaching

The multitude of pedagogic and stylistic approaches for teaching immunology is revealed in articles describing undergraduate immunology classes. The role of storytelling employs the device of micro stories- short narratives designed to harness the affective and cognitive benefits of storytelling in minimal amounts of time (Lukin). These narratives employ concepts of neuroscience to enhance memory formation and emphasize how these can be incorporated to address the diversity of students. On a more global level, the paper by Bruns et al. explores the comparison of immunology as a discipline to neuroscience, which have many intellectual similarities to immunology but is significantly more highly represented in the number of undergraduate majors and discusses the potential reasons why this might occur. This article, as well as a recently published paper (2), details how undergraduate teaching and the curriculum might be changed to enhance Immunology a paradigm for interdisciplinary courses. Chatterjea discusses teaching immunology as a liberal art and emphasizes how immunology is not a single story. Her perspective is captured by the quote that many immunologists would subscribe to “I am grateful that immunology—the beautiful, maddening, messy field that it is—keeps me humble and honest about the work I really want to do with my students and the way in which I want to do it.” It reflects both the difficulty and the optimism about teaching immunology that many Immunology professors have. Rawlings describes how to incorporate primary literature at the undergraduate level. While this often occurs at the graduate level, he provides a concrete framework of how to incorporate original articles into undergraduate courses. In a related vein, Stranford et al. offer a compendium of strategies to increase active learning and engagement based on their collective experience of over 90 years of teaching. Acknowledging that no one could be expected to incorporate all of these approaches, they expertly describe multiple strategies that can be employed to promote active learning. Collectively, these papers indicate that whatever strategy one takes, it is important to make a commitment to your chosen approach which needs to be clearly articulated and defined.

## Challenges of Lab Courses

Many immunologists believe one of the most effective ways to truly learn immunology is thorough lab work. However, lab courses are particularly challenging as outlined in the 3 papers in this Research Topic (Garrison and Bupp; de Vries. et al.; Demaria et al.). They are labor -intensive, require laboratory space rather than classrooms or lecture halls, and often demand sophisticated instruments and specialized supplies. From an institutional standpoint they are extremely expensive. As a result, many schools have greatly reduced the number, or even eliminated, lab courses. Moreover, they require experiments to be done in rather strict timelines (such as 4 hour blocks) compared to “real research” in which the experiments rather than the schedule dictate the experimental design. This is true not only in the actual blocks of lab time but also in the length of the entire class (de Vries. et al.). Concrete strategies to overcome the many obstacles and successfully deliver lab courses are presented (Garrison and Bupp; de Vries. et al.; Demaria et al.) are presented. Ways to provide a realistic experience with an inquiry-based lab experience is illustrated in Demaria et al. In these articles there is an emphasis on how the experiments can be used to help understand the theoretical underpinnings and underlying immunological basis of the experiments as well as their practical applications.

## Immunology for Students in Healthcare Professions

Teaching immunology for the healthcare professions can have some advantages compared to most undergraduate courses. Acknowledging there is a wide range of students even among health care pre-professionals, they are often more self-motivated than undergraduates, and in many cases have a stronger background in basic sciences, having already mastered many concepts and techniques in genetics, microbiology, and biochemistry. The stronger background of these students allows the teacher to use their knowledge of these disciplines in introducing immunologic concepts. On the other hand, pre-professionals often have a bias about what they believe is important and are resistant to learning about topics they perceive are not applicable or represent rare cases (Karim). They are particularly motivated by short-term goals such as professional licensure or other test requirements which often drive curriculum. Strategies that help balance these factors are outlined in Haidaris and Frelinger which shares some lessons for teaching medical students in a multidisciplinary medical school course. Teaching medical students or professionals through case studies to promote student engagement with case based scenarios is the topic of two of the articles (Karim) and (Novack). The relative merits of using uncommon cases where the underlying immunology is very clear *versus* more common cases where the immunology is more complex is also addressed. Additional approaches of how to teach immunology to health care professionals include team-based approaches (James et al.) and Just-in-time teaching (JiTT) (Madiraju et al.). These approaches emphasize student engagement and the use of pre-class preparation, and give concrete guidance as how to develop these approaches to promote active learning.

## Special Challenges in Non-Traditional Students and Audiences

Many of the articles in this Research Topic acknowledge that immunology is a complicated field with an arcane vocabulary and a steep “learning curve”. Moreover, it is often non-intuitive while also requiring fundamental knowledge from many other fields. Illustrating immunology concepts is particularly challenging when the audience has little or sometimes no formal training in basic science such as lay audiences. Indeed, such “teaching”, is often considered outside the normal teaching endeavors of immunologists, although it is crucial in maintaining support for scientific research. Ellis and Pennell discuss their efforts to reach general audiences using cancer immunotherapy as the paradigm. How to provide immunology teaching poses other challenges in resource-constrained countries. Kabelitz et al. provide a framework to deliver immunology concepts and information using a combination of online learning modules in conjunction with information presented in an intense course. Importantly, they also provide resources that can be used after the course ends so that individuals can remain current in a rapidly evolving field. In related topics, two of the papers discuss some of the particular issues with attracting and retaining underrepresented groups which has received national attention (3). Smolock and Robert discuss how they restructured their pipeline research to increase trainee success and retention. These include incorporating the many disciplines that are relevant to immunology as well as structural aspects such as skill-building workshops and better cross campus integration with student diversity groups and the Office of Diversity and Inclusion. Riestra et al. discuss high level pedagogy that outline barriers to equitable achievement, including avoiding stereotypes and emphasizing values, relevance, clear paths to achievement, and mastering vocabulary.

## COVID-19 Effects of Teaching Immunology

It would be remiss to conclude this Research Topic without mentioning the effect of COVID-19 on teaching Immunology. The tragic human and economic cost of the pandemic, and the impressive success of vaccine strategies against it, have highlighted the significance of immunology as a science with direct, practical impact on society. Complex concepts such as antibody titers, neutralization assays, passive immunity using monoclonal antibodies, booster shots, cross reactivity, cytokine storms, and T cell memory have started to enter the general vocabulary of the entire population. At the same time, the need to fight public misinformation from vaccination opponents leading to widespread vaccine hesitancy highlights the great opportunity and challenge of explaining immunology in a way that the general public can understand. Indeed, it has become evident that the general population and even scientists in other disciplines often have fundamental misunderstanding of immunology and the immune response. The articles reflect teaching approaches before the COVID crisis, but like almost all aspects of society, the teaching of immunology has changed dramatically in the past 18 months. These changes range from shifting emphasis on specific teaching topics (e.g., anti-viral responses, immunopathology, vaccine mechanisms), to the adoption of new approaches and technological platforms for remote learning. Which of these changes will endure remains to be seen. However, at the same time, it is clear that certain established principles and methods in teaching Immunology remain very relevant, reflecting not only key ideas and concepts, but also the fundamentals of all teaching: student engagement, active learning, and enthusiasm.

## Author Contributions

All the authors contributed equally to this editorial. All authors contributed to the article and approved the submitted version.

## Conflict of Interest

The authors declare that the research was conducted in the absence of any commercial or financial relationships that could be construed as a potential conflict of interest.

## Publisher’s Note

All claims expressed in this article are solely those of the authors and do not necessarily represent those of their affiliated organizations, or those of the publisher, the editors and the reviewers. Any product that may be evaluated in this article, or claim that may be made by its manufacturer, is not guaranteed or endorsed by the publisher.
